# Immune-related adverse events in patients with preexisting myasthenia gravis and thymoma following immune checkpoint inhibitor treatment: a retrospective, observational study

**DOI:** 10.3389/fimmu.2026.1635001

**Published:** 2026-03-10

**Authors:** Chao Sun, Rongjing Guo, Xunliang Yin, Lanlan Feng, Yize Guo, Yueliang Xu, Sijia Hao, Xiaoxi Huang, Na Song, Ting Gao, Jie Liu, Li Gong, Jiayu Lu, Qiang Lu, Yongan Zhou, Ting Chang

**Affiliations:** 1 Department of Neurology, Tangdu Hospital, The Fourth Military Medical University, Xi’an, Shaanxi, China; 2 Department of Thoracic Surgery, Tangdu Hospital, The Fourth Military Medical University, Xi’an, Shaanxi, China; 3 Department of Pathology, Tangdu Hospital, The Fourth Military Medical University, Xi’an, Shaanxi, China; 4 Department of Critical Care Medicine, Tianjin Medical University General Hospital, Tianjin, China; 5 Basic Medical College, The Fourth Military Medical University, Xi’an, Shaanxi, China

**Keywords:** germinal centers, immune checkpoint inhibitors, immune-related adverse events, myasthenia gravis, thymoma

## Abstract

**Objective:**

Immune checkpoint inhibitors (ICIs) can induce immune system activation and cause immune-related adverse events (irAEs). This study aimed to assess the incidence and management of irAEs in thymoma patients with preexisting MG who received ICI therapy.

**Methods:**

This was a retrospective observational cohort study. From September 2018 to May 2024, 12,916 patients received ICI therapy at our hospital. Among them, six patients with preexisting MG and thymoma (MGT) received ICI treatment, and ten thymoma patients without MG (TOMA) served as controls. irAEs, MG flares, and treatment outcomes were primarily assessed through retrospective review of medical records. Anti-acetylcholine receptor antibody (AChR-Ab) levels and pathological thymoma tissue features were analyzed to explore the potential mechanisms underlying the irAEs.

**Results:**

Compared with TOMA patients (n=10), all MGT patients (n=6) had grade 3 or higher irAEs (p=0.034) and experienced ICI-induced myocarditis (p=0.011). All MGT patients experienced symptom exacerbation, including a myasthenic crisis. MGT patients who received immunosuppressive agents before ICI therapy and those who received both steroids and intravenous immunoglobulin (IVIG) during irAE occurrence had better outcomes. AChR-Ab levels markedly increased one month after the onset of irAEs. Furthermore, two TOMA patients with germinal centers (GCs) in their thymus tissues had severe irAEs, whereas two without GCs had no irAEs.

**Conclusion:**

In this study, irAEs were common and severe in patients with preexisting MG and thymoma following ICI therapy. Pretreatment immunosuppressive therapy was associated with better clinical outcomes. The presence of GCs in thymoma patients without MG may serve as a predictive biomarker for the occurrence of irAEs.

## Introduction

Immune checkpoint inhibitors (ICIs) have transformed the therapeutic landscape of numerous cancers by activating the immune system to target tumor cells. However, this nonspecific immune activation frequently leads to off-target immune-related adverse events (irAEs). Patients with preexisting autoimmune diseases (PADs) were initially largely excluded from ICI clinical trials due to concerns about autoimmune disease flares and elevated irAE incidence (1). Real-world evidence has shown that certain PAD patients—such as those with rheumatoid arthritis or systemic sclerosis—exhibit relatively mild irAE profiles during ICI treatment (2–4). In contrast, other preexisting inflammatory conditions are associated with a higher risk of severe irAEs (5).

As a prototypical antibody-mediated, T-cell-dependent autoimmune disorder, myasthenia gravis (MG) causes neuromuscular junction dysfunction. Approximately 85% of patients with MG have detectable AChR antibodies. Notably, up to 10–15% of AChR antibody-positive MG patients harbor thymomas, and 30–45% of thymoma patients develop MG (6, 7). Existing studies on ICI therapy in patients with preexisting MG have primarily focused on melanoma (8), and MG has been linked to a higher incidence of severe irAEs following ICI treatment compared to other preexisting autoimmune neurological disorders (9). Yet, the safety of ICIs and management of irAEs in thymoma patients with preexisting MG remain poorly characterized.

Refractory or recurrent thymomas show limited responsiveness to radiotherapy and chemotherapy (10). Additionally, corticosteroid-sparing immunosuppressive (CSIS) medications used for MG management increase the risk of extrathymic malignancies, including cancers of the digestive tract, male genital organs, and skin (11). While ICIs represent a critical therapeutic option for unresectable tumors, current guidelines caution against their use in patients with MG and/or thymoma due to the high risk of irAEs (12).

A recent study highlighted the susceptibility of thymoma patients to irAEs. However, pretreatment data on the association between AChR Ab status and myocarditis in this population are scarce. While most MG patients with thymoma (MGT) test positive for AChR Abs, more than half of AChR Ab-positive individuals do not have clinical manifestations of MG (13), leaving the mechanism underlying the predisposition to myocarditis in thymoma without MG (TOMA) patients unclear. Importantly, prior research has not separately or systematically analyzed clinical outcomes in patients with preexisting MG and thymoma initiating ICI therapy (9, 14).

MGT is characterized by ectopic germinal centers (GCs) in thymoma tissue and adjacent adipose tissue (15), with approximately 33.3% of GC-positive thymoma patients developing MG, whereas only 2.3% of GC-negative patients develop MG (16). These ectopic GCs, which are composed of B cells and T follicular helper cells, produce pathogenic AChR Abs that drive MG pathogenesis. Although AChR Abs have been proposed as biomarkers for irAEs in thymoma, the specific impact of preexisting MG on irAE severity during ICI therapy remains uninvestigated.

Given thymoma patients’ inherent predisposition to irAEs, we included a control cohort of thymoma patients without MG (TOMA group) to isolate the additional risk specifically attributable to preexisting MG, while controlling for the background risk associated with thymoma and ICI therapy. In this study, we aimed to 1) compare the incidence of severe irAEs between MGT and TOMA patients receiving ICIs; 2) characterize management strategies for MGT and longitudinal changes in AChR Ab levels during the acute and chronic irAE phases; and 3) explore the relationship between thymoma histopathology (particularly the presence of GCs) and irAE manifestations. These investigations aim to refine monitoring strategies and elucidate the mechanisms underlying irAEs in MGT and TOMA patients.

## Materials and methods

### Study design and participants

This retrospective, observational study included all preexisting MG patients who received ICI therapy from September 2018 to May 2024 at Tangdu Hospital of The Fourth Military Medical University. All six patients with myasthenia gravis who received ICIs had unresectable thymoma without other concurrent tumors, which was the indication for ICI treatment. Additionally, ten patients with unresectable thymoma but without MG were included as control subjects. Two patients with both preexisting MG and unresectable thymoma received immunotherapy at outside hospitals. The reasons for thymoma unresectability are detailed in **Supplementary Table 1**. The preliminary diagnosis of thymoma, also known as thymic epithelial tumor (TET), was based mainly on chest CT and contrast-enhanced chest CT. The histopathological diagnosis of the samples was based on the World Health Organization (WHO) guidelines and confirmed by two pathologists. The first prescription date of ICI therapy was defined as the index date. ICI therapy drugs targeting PD-1 (pembrolizumab, camrelizumab, tislelizumab, sintilimab, and toripalimab) and PD-L1 (cejemly) were administered.

MG was diagnosed on the basis of a combination of fluctuating muscle weakness and fatigability, with at least one of the following criteria fulfilled: (a) AChR Ab positivity, (b) a positive response to the neostigmine test, and (c) an abnormal decrease in low-frequency repetitive nerve stimulation (RNS) tests. Patients who developed MG following ICI therapy were excluded. The distribution and severity of myasthenic weakness were assessed using the Myasthenia Gravis Foundation of America (MGFA) classification and the Myasthenia Gravis Activities of Daily Living (MG-ADL) scale within 1 week before ICI therapy. In this study, myasthenic crisis was defined as respiratory failure requiring noninvasive ventilation or endotracheal intubation. Myasthenic crisis of respiratory failure is caused mainly by neuromuscular junction disorders (17, 18). The clinical data of all patients were obtained from medical records.

### Outcomes

The primary outcome of this study was the safety of ICIs in thymoma patients with or without MG. The safety of ICIs was gauged on the basis of the incidence of grade 3+ irAEs after one month of ICI therapy. Grade 3+ irAEs were defined as severe according to the Common Terminology Criteria for Adverse Events (version 5.0). Hepatitis severity was graded mainly on the basis of the serum levels of alanine aminotransferase (ALT) and aspartate aminotransferase (AST). Autoimmune hemolytic anemia was graded primarily on the basis of the hemoglobin level. Thyroiditis was diagnosed through the evaluation of thyroid function test results. Myocarditis was primarily diagnosed through the evaluation of electrocardiogram findings, myocardial injury biomarkers, echocardiogram results, and brain natriuretic peptide (BNP) levels, with confirmation by a cardiology expert.

Symptoms of MG flares were identified using the MGFA classification system and the MG-ADL scoring system. MGFA classification and MG-ADL scores were obtained one week prior to the initiation of ICI treatment, at the time of the most severe irAEs, and at 6 months and 1 year after irAE occurrence. The dosages of pyridostigmine, glucocorticoids and immunosuppressants in patients with MG were obtained via electronic medical records and face-to-face or telephone follow-up.

### Histological assessment

Thymoma tissues were obtained prior to the initiation of ICI therapy. For some patients who underwent thymectomy at external hospitals, thymoma tissue samples were unavailable. For others, thymoma tissues were obtained via puncture biopsy; however, the samples were insufficient for hematoxylin and eosin (HE) staining and immunohistochemistry (IHC) analysis. The pathological characteristics of the remaining eight patient samples, excluding those of the puncture biopsy specimens, were analyzed via IHC. All the samples were fixed in 10% neutral formalin and embedded in paraffin. The diagnoses were confirmed by two expert pathologists, who were blinded to the clinical data of patients receiving ICI therapy and who performed the evaluations according to the WHO classification, including determination of the presence of GCs. Sections were stained with HE and then examined via light microscopy to observe the structural characteristics of the GCs. Further analysis of the CD21 marker was conducted via IHC to determine the presence of GCs (16). CD21 is expressed predominantly on the surface of mature B cells and follicular dendritic cells, two major components of GCs. IHC was performed using primary antibodies against rabbit CD21 (EP64; MXB, China) according to the manufacturer’s instructions.

### AChR testing

Peripheral blood samples were collected from patients with MG in stable condition within 2 years before ICI initiation, during the occurrence of irAEs, and one month after the irAEs. The AChR Ab titer in the serum was quantified via a radioimmunoprecipitation assay (RIPA), and the AChR Ab titer was assessed by measuring the percentage loss of binding sites for ^125^I-bungarotoxin. The standard procedure was performed with the AChR Autoantibody RIPA Kit from RSR (RSR Ltd., UK) according to the manufacturer’s instructions. A test result exceeding 0.4 nmol/L was considered a positive indication.

### Statistical analysis

Continuous variables were expressed as the means ± standard deviations (SDs) when normally distributed, whereas the median and interquartile range (IQR) were used for nonnormally distributed continuous variables. Categorical variables are expressed as frequencies (n) and percentages (%). To compare continuous variables between MGT and TOMA patients, the Mann-Whitney U test was used for nonnormally distributed data. Student’s t test was used to compare normally distributed continuous variables between two groups. For categorical variables, differences between the MGT and TOMA groups were compared using Fisher’s exact test in IBM SPSS Statistics 20. A two-tailed p-value < 0.05 was considered statistically significant. GraphPad Prism 8 software (GraphPad Software, Inc., USA) was used to generate the figures.

## Results

### Baseline characteristics

A total of 12,916 patients who received ICI therapy for cancer treatment at a single hospital center between September 2018 and May 2024 were screened. Among them, six patients had a preexisting diagnosis of MG before the initiation of ICI therapy and presented with unresectable thymoma (MGT group). Furthermore, 10 patients with unresectable thymoma without MG (TOMA group) were included as controls (**Supplementary Figure 1**).

The median age of the 16 study participants was 55 years (interquartile range [IQR] 46.5–56.75), and the age at thymoma onset was 49.8 ± 11.2 years. Among the 16 participants, 7 (43.8%) were female, and 9 (56.3%) were male (**Table 1**). The median body mass index (BMI) was 22.6 (IQR 21.93–25.25) in both groups. PD-1 inhibitors were the most common ICI target (6 [100%] of 6 MGT patients *vs.* 9 [90%] of 10 TOMA patients). Thymomas were mainly grade B2 or B3 in the TOMA group and B2 in the MGT group. The other baseline characteristics of the patients are presented in **Supplementary Table 1**.

**Table 1:** Baseline characteristics.

Variables	TOMA (n=10)	MGT (n=6)	*p* value
Age (years)	48.5 (43.5-59.5)	55.5 (55–56)	0.425
Sex			
Male	5 (50%)	2 (33.3%)	0.633
Female	5 (50%)	4 (66.7%)	
Body-mass index (kg/m2)	22.8 (22.4-28.1)	22.3 (21.3-24)	0.326
onset age of thymoma (years)	43.3	53.7	0.13
ICI therapy			
PD-1	10 (100%)	5 (83.3%)	
PD-L1	0 (0%)	1 (16.7%)	
CTLA-4	0 (0%)	0 (0%)	
Combination	0 (0%)	0 (0%)	
WHO classification			
AB	1(10%)	0(0%)	
B1	0(0%)	1(16.7%)	
B2	5(50%)	5(83.3%)	
B3	4(40%)	0(0%)	
Masaoka stage			
III	3 (30%)	1 (16.7%)	
IVa	4 (40%)	2 (33.3%)	
IVb	3 (30%)	3 (50%)	

MGT, Myasthenia Gravis with thymoma; TOMA, Thymoma without myasthenia gravis; ICI, immune checkpoint inhibitor.

### irAE grade and type

Among the patients who underwent ICIs therapy for thymoma, all 6 (100%) MGT patients experienced irAEs of all grades compared with 5 (50%) TOMA patients. Severe irAEs (grade 3 or higher) occurred in all 6 (100%) MGT patients, whereas 4 (40%) TOMA patients experienced severe irAEs (*p* = 0.034). In this study, 6 (100%) MGT patients and 3 (30%) TOMA patients developed myocarditis (*p* = 0.011). There were 3 (50%) and 3 (30%) grade 5 irAEs (death) due to myocarditis in the MGT and TOMA groups, respectively (**Table 2**).

**Table 2:** Severity and type of irAEs.

irAEs	TOMA	MGT	p value
All irAEs grades	5 (50%)	6 (100%)	0.093*
Grade 3+	4 (40%)	6/6 (100%)	0.034*
Grade 4+	4 (40%)	6/6 (100%)	0.034*
Grade 5	4 (40%)	3 (50%)	1
All irAEs types			
one or more irAEs	5 (50%)	6 (100%)	0.093*
two or more irAEs	5 (50%)	5 (83.33%)	0.307
three or more irAEs	1(10%)	4 (66.67)	0.036*
four or more irAEs	0 (0%)	3 (50%)	0.036*
Specific irAE types			
Myocarditis (grade 3+)	3 (30%)	6 (100%)	0.011*
Hematological (cytopenia, autoimmune hemolytic anemia) (grade 3+)	1 (10%)	0 (0%)	0.625
Myasthenia gravis (grade 3+)	0 (0%)	6 (100%)	
Hepatitis (grade 3+)	3 (30%)	4 (66.67%)	0.302
Thyroiditis	5 (50%)	3 (50%)	1
irAEs after one cycle	5 (100%)	6 (100%)	0.093
The time to disease onset (days)	13.6	14.67	0.874

irAEs, immune-related adverse events; MGT, Myasthenia Gravis with thymoma; TOMA, Thymoma without myasthenia gravis. *p< 0.05.

ICI drugs can also cause MG-like syndromes, characterized by typical MG symptoms such as ptosis, dysphagia, and limb weakness (14). However, no patients in the TOMA group developed clinically apparent MG-like syndromes. In the patients with MG, the condition recurred or exacerbated after ICI therapy. Hepatitis occurred in 4 (67%) patients in the MGT group and 3 (30%) patients in the TOMA group. Thyroid dysfunction was observed in 3 (50%) MGT patients and 5 (50%) TOMA patients. All patients with irAEs developed these symptoms after the first ICI dose, and there was no statistically significant difference in the timing of the first irAEs between the MGT and TOMA groups (*p* = 0.874). Individual patient clinical data are provided in **Supplementary Table 1**.

### Myasthenia gravis flare and treatment

In the MGT group, 3 (50%) patients developed grade 4 irAEs, and 3 (50%) patients developed grade 5 irAEs (death) due to myocarditis. The MGT patients were divided into nonsurviving or surviving groups according to their outcomes (**Table 3**). Among the patients, 3 (50%) were female. The median patient age was 48.5 years (IQR 43–64.5). The median age of MG onset was 43.5 years (IQR 29.5–57), and the median disease duration of MG was 5.5 years (IQR 3–7). The MG-ADL score, MGFA classification, and symptoms of stability indicated that all patients with MG had mild symptoms and that their disease was in a well-controlled state. The pathological grade of the thymoma was B2 in 5 MG patients and B1 in 1 patient, which is consistent with the fact that MG patients often have B-type thymoma. In the nonsurviving group, all patients had experienced a previous myasthenic crisis (MC), whereas only 1 patient in the surviving group had experienced a previous crisis. In this study, all MGT patients developed myocarditis after receiving initial ICI therapy, and all deaths were attributed to myocarditis.

**Table 3:** irAEs and treatment of MG.

Variables	Surviving group			Nonsurviving group		
MG patients no	No 1	No 2	No 3	No 4	No 5	No 6
irAEs grade	4	4	4	5	5	5
Baseline characteristics						
age (year)	60s	20s	40s	40s	60s	50s
age at onset (year)	60s	10s	40s	40s	50s	40s
The duration of MG (years)	3	6	5	1	8	8
AChR Ab	Yes	Yes	Yes	Yes	Yes	Yes
Symptoms within 3 months	Stable	Stable	Stable	Stable	Stable	Stable
MG-ADL before ICI	0	2	0	2	0	2
MGFA before ICI	PR^a^	1a	PR^a^	1b	CSR^b^	1a
The dose of pyridostigmine mg/day	0	180	0	120	0	480
The dose of prednisone acetate mg/day	0	10	0	0	0	0
The dose of azathioprine mg/day	100	0	75	0	0	0
The dose of tacrolimus mg/day	0	0	0	0	0	0
Breath difficult history	Yes	Yes	No	Yes	Yes	Yes
MC history	No	Yes	No	Yes	Yes	Yes
Histologic analysis (WHO classification)	B2	B2	B2	B1	B2	B2
irAEs and MG flare						
ICI cycle	1	1	1	1	1	1
Myocarditis	Yes	Yes	Yes	Yes	Yes	Yes
Myocarditis grade	4	4	4	5	5	5
ADL score during irAEs	17	17	15	14	16	12
MGFA score during irAEs	V	V	V	V	V	V
Time to irAEs (day)	30	21	7	3	5	22
Hepatitis (grade 3+)	no	no	yes	yes	yes	yes
Treatment						
IVIG	Yes	Yes	Yes	No	Yes	No
Corticosteroids	Yes	Yes	Yes	Yes	No	Yes

MG, Myasthenia Gravis; irAEs, immune-related adverse events, AChR Ab, anti- acetylcholine receptor antibody, MG-ADL, Myasthenia Gravis Activities of Daily Living; MGFA, Myasthenia Gravis Foundation of America; MC, Myasthenic crisis; ICI, immune checkpoint inhibitor.

MGFA postintervention status for asymptomatic patients: ^a^pharmacologic remission (PR) and ^b^complete stable remission (CSR).

To achieve long-term stabilization of MG symptoms, patients in the surviving group were on low-dose oral steroids or immunosuppressants prior to the use of ICI drugs. In the surviving group, 3 (100%) MG patients received a combination of steroids and IVIG therapy during the early stage of irAEs. In the nonsurviving group, 2 patients exclusively received steroid therapy, whereas 1 patient received only IVIG therapy. For MGT patients with irAEs, early use of IVIG combined with steroids may lead to better outcomes than steroids or IVIG alone (**Table 3**).

### Long-term follow-up of MG patients

**Figure 1:**
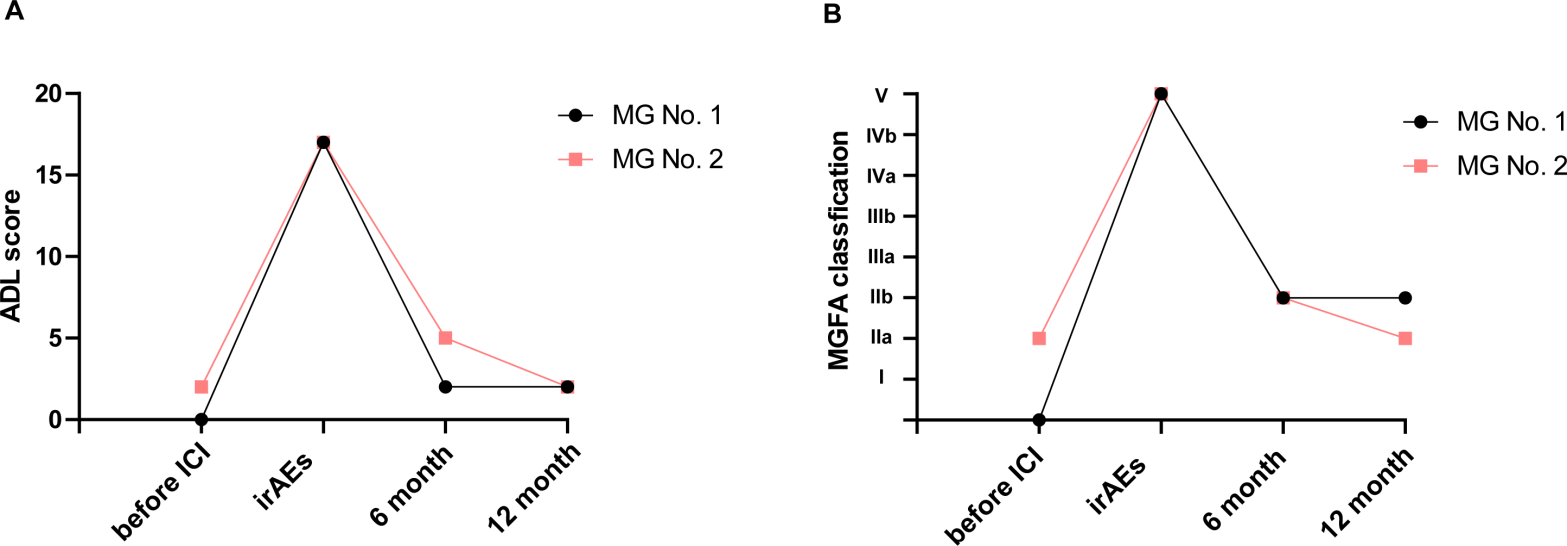
MG symptom flares. The severity and distribution of MG symptom flares were based on the MG-ADL score **(A)** and MGFA classification **(B)**. Patients No. 1 and No. 2 were followed up from one week before ICI, during the irAE period, and then at 6 months and 12 months after the onset of irAEs.

Both MG patients No. 1 and No. 2 were administered pyridostigmine and immunosuppressants (either steroids or azathioprine), and the drug doses were gradually decreased according to the symptoms. The symptoms of MG generally improved to a mild and stable state on the basis of the MG-ADL score and MGFA classification after 6 months (**Figure 1**). In addition, MG patient No. 2 was treated with oral anlotinib hydrochloride at a dosage of 12 mg/day for 6 months, and the thymoma showed a partial response on chest CT (data not shown).

### Changes in the AChR Ab level

**Figure 2:**
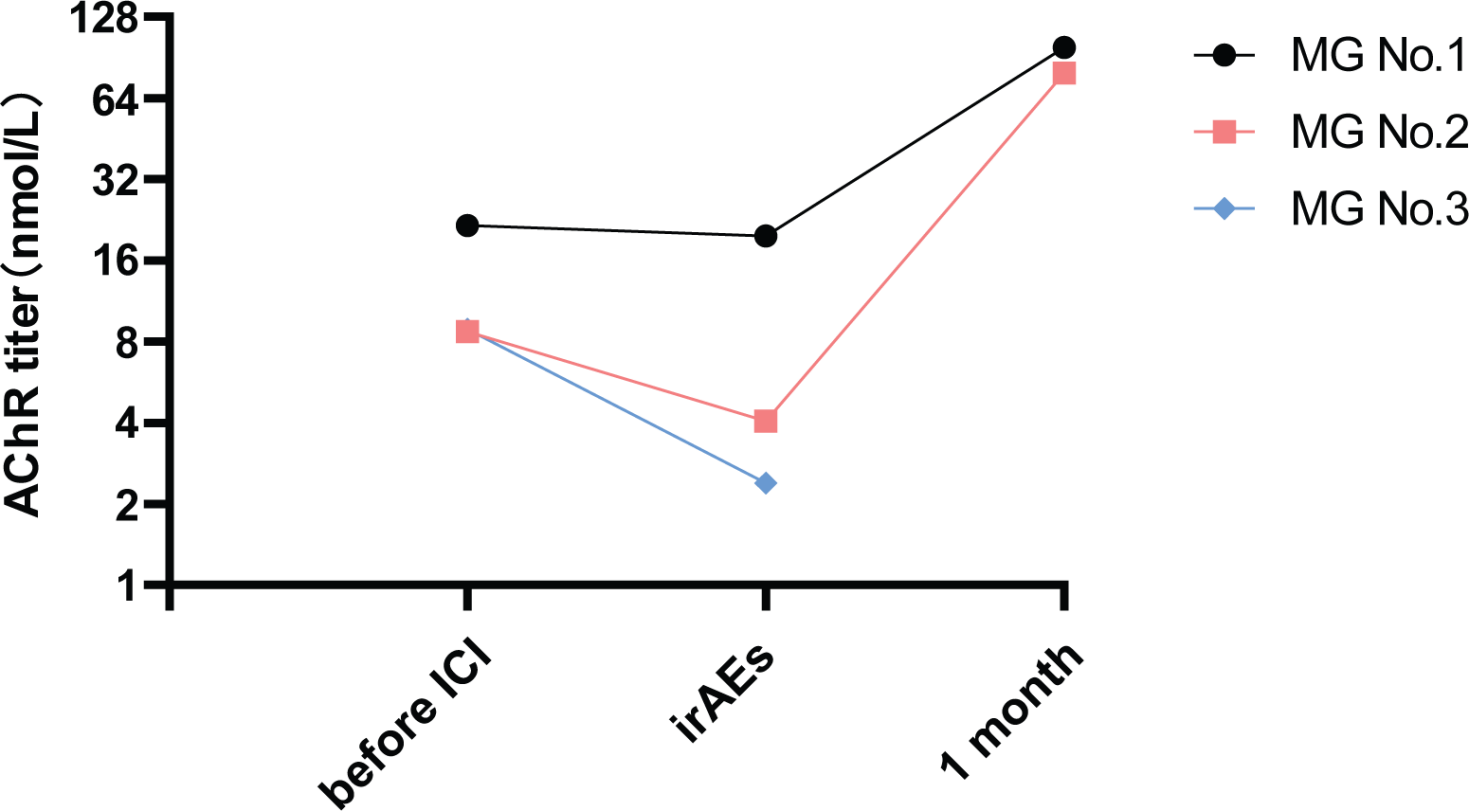
Changes in the AChR Ab level before and after the onset of irAEs. The AChR Ab levels for MG patients No. 1, No. 2, and No. 3 were measured before ICI treatment, at the onset of irAEs and at 1 month after irAE onset. Patient No. 3 was lost to follow-up after the irAE period.

Compared with pre-ICI AChR Ab levels, no significant elevation was observed at irAE onset; two patients even showed decreased levels. In addition, the levels of AChR antibodies increased more than 10-fold at 1 month post-irAE in patient No. 1 and patient No. 2, but the symptoms of MG in these two patients improved significantly (**Figure 2**).

### Thymic immune microenvironment and irAEs

**Figure 3:**
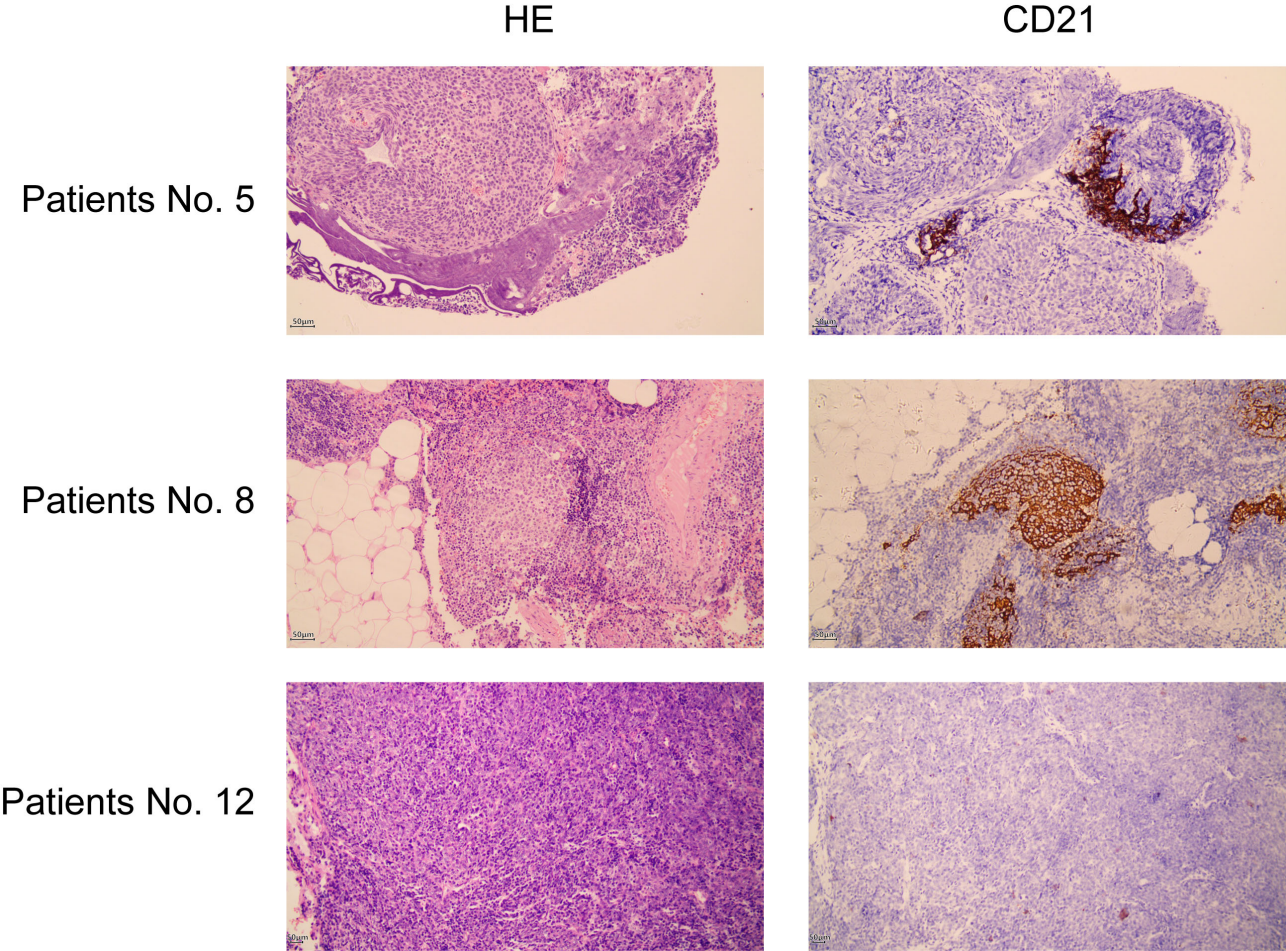
Representative images of immunohistochemical staining of the thymoma. GCs were identified by HE staining and CD21 staining. CD21 expression in GCs indicates the presence of follicular dendritic cells. Patient No. 5 was an MGT patient with two GCs. Patient No. 8 was a TOMA patient with irAEs, and four GCs were present. Patient No. 12 was a TOMA patient without irAEs and without GCs (scale bar= 50 μm).

MG often coexists with thymoma, and GCs in thymomas are the main source of pathogenic AChR Abs. In the present study, thymoma tissue was available for only 4 patients with MG, and GCs were found in 3 patients with MG by HE staining and IHC. In the TOMA group, thymoma tissues were obtained from 2 patients with irAEs and 2 patients without irAEs (**Supplementary Table 2**). We detected GCs in patients with irAEs, whereas no GCs were detected in patients without irAEs (**Figure 3**). Therefore, the presence of GCs in a thymoma may be closely associated with irAE development.

## Discussion

MG is a rare, prototypical autoimmune neuromuscular junction disease that is frequently comorbid with thymoma. This study focuses specifically on the management of irAEs in thymoma patients with preexisting MG receiving ICI therapy. Our findings highlight an elevated risk of severe irAEs (particularly myocarditis and myasthenic crisis) in this population. The maintenance of immunosuppressants prior to ICI initiation, combined with steroid and IVIG combination therapy following irAE onset, may be critical for improving clinical outcomes in MG patients. The exacerbation of MG by ICIs is not primarily mediated by the activation of B cells but may be directly associated with the activation of AChR-specific T cells. The presence of ectopic GCs is closely linked to the occurrence of irAEs in thymoma patients with or without MG.

ICIs are administered to patients with MG, mainly those with unresectable thymoma. In a retrospective study of low-dose nivolumab administered for metastatic thymic epithelial tumors, 2 out of 8 patients had preexisting MG, and 1 patient was positive for AChR Ab but had no MG symptoms. The two MG patients experienced severe (grade 4) irAEs after 1 cycle, and the AChR Ab-positive patient showed a grade 1 response after 3 cycles (19). In another study, 3 patients with preexisting MG, all of whom had MGFA type 1, had not received immunosuppressants for at least 1 year before ICI therapy. All three patients with MG developed grade 3 or higher irAEs after the first dose of ICIs (10). However, these studies lacked comparative analyses between thymoma-associated MG and thymoma-only cohorts, and the sample sizes were limited (<3 MG cases).

Compared with other autoimmune diseases, MG is rare, with an annual incidence of approximately 0.68 per 100,000 individuals in China (20). In our study, 6 MG patients with thymomas who had received ICI drugs were included, making this the largest single-center study to date. During the same period, 10 patients with thymoma alone were included as the control group. All 6 MG patients experienced an exacerbation of MG symptoms and myocarditis, with grade 4 or above irAEs. In the thymoma group, 4 out of 10 patients (40%) experienced grade 4 or higher irAEs, which is in line with other reports in the literature (14). Therefore, despite the limited sample size of 6 MG patients with thymoma and 10 patients with thymoma, our data support the notion that MG patients with thymoma are at high risk of irAEs when they are receiving ICIs.

ICI-induced myocarditis occurs in less than 1% of patient cases, excluding thymoma patients, and the mortality rate is 30–50% (21). For patients with thymoma, the incidence of ICI-induced myocarditis ranges from 16% to 29%, with a mortality rate of up to 64% (14). Myocarditis, a rare but fatal irAE (21), was also the primary cause of mortality in our cohort. In murine models, infiltrating CD8+ T cells recognize α-myosin antigens, and α-myosin-expanded TCRs are present in inflamed cardiac and skeletal muscle in patients with ICI-induced myocarditis (22, 23). Since alpha-myosin is not expressed in the thymus, the specific T cells involved in thymoma-induced autoreactive myocarditis need to be further determined. One study reported that 11–13% of MG patients with thymoma also had an autoimmune disease; notably, neurological autoimmune diseases were frequently paired with thymoma or thymic hyperplasia (24). These findings indicate that GCs in the thymoma are closely related to autoimmune disease. Therefore, ectopic GCs in MGT patients may contain autoreactive T cells and B cells that target other tissues (non-AChR) and represent abnormalities in the immune microenvironment in thymoma tissue. Consistent with this, both MGT patients and TOMA patients with GCs developed myocarditis in our study, indicating abnormalities in the immune microenvironment of thymomas with GCs. We propose that ectopic GCs in thymomas harbor autoreactive T/B cells that target non-AChR tissues and that ICI-induced T-cell activation may affect cardiac/skeletal muscle.

The specific mechanism underlying ICI-induced exacerbation of MG remains incompletely understood, and this exacerbation is not solely dependent on B-cell activation, as patients show no significant elevation in autoantibody levels during the occurrence of irAEs. Instead, it is more likely attributed to the extensive disruption of immune tolerance by ICIs. By blocking the CTLA-4 and PD-1/PD-L1 pathways, ICIs nonspecifically abrogate the inhibition of CD4+ and CD8+ T-cell subsets, leading to multidimensional immune dysregulation. On the one hand, they can directly activate preexisting acetylcholine receptor (AChR)-specific CD8+ cytotoxic T cells, which then target and attack neuromuscular junctions. This mechanism is supported by studies using the experimental autoimmune myasthenia gravis (EAMG) model, in which depletion of CD8+ T cells significantly alleviated MG symptoms (25). Additionally, research has confirmed that the proportion of central memory CD8+ T cells (Tcm) is increased while the proportion of effector memory CD8+ T cells (Tem) is decreased in MG patients (26). On the other hand, ICIs concurrently activate CD4+ T helper cells, particularly Th1 and Th17 subsets, which secrete cytokines, including IL-2 and IFN-γ (27). These cytokines not only increase the cytotoxicity of CD8+ T cells but also drive B-cell proliferation, differentiation, and autoantibody production (10, 27). Furthermore, ectopic germinal centers in thymoma tissues are enriched in autoreactive T and B cells (15), and ICIs can significantly increase the activation efficiency of these cells, further exacerbating immune microenvironment dysfunction. In summary, ICI-related MG exacerbation is a multidimensional synergistic outcome. These findings underscore the need for further mechanistic investigations, with a particular focus on the effects of ICIs on ectopic germinal centers within thymomas.

In the case of ICI drug-induced symptom exacerbation in patients with preexisting MG, those treated with combined IVIG and steroids had more favorable outcomes than those receiving steroids or IVIG monotherapy did. Similarly, for ICI drug- induced MG, IVIG or PLEX may lead to better outcomes than steroids alone. Two patients with preexisting MG did not experience worsening symptoms, and both were given 10 mg of corticosteroids before ICI therapy. Six patients with ICI-induced MG received ICI treatment again, and the symptoms of MG did not worsen (8). This may have occurred because all 6 patients received immunosuppressive treatment before the readministration of ICI treatment. On the basis of our findings, we suggest that even with stable MG symptoms, maintenance of low-dose prednisone and/or immunosuppressive agents prior to ICI therapy may significantly reduce ICI-induced mortality. Given the high risk of irAEs, consultation with a neurologist is recommended for preexisting MG patients before ICI therapy is initiated.

Currently, considering the high incidence and severity of irAEs in thymoma patients, ICI therapy for these patients is not recommended. However, some thymoma patients do not experience irAEs after receiving ICIs, and some may even experience a partial response (PR) (10). Similarly, in our study, we found that 5 TOMA patients (50%) did not experience significant irAEs. Therefore, identifying the appropriate population is crucial for providing ICI treatment to thymoma patients. Fenioux et al. proposed that the AChR Ab level could be a biomarker for irAEs in thymoma patients receiving ICI therapy (14). However, the origin of AChR Abs (thymus, peripheral blood, or other sources) and the timing of their production remain unclear. In patients with MG, the levels of AChR Ab are closely associated with the number of GCs. TOMA patients are also positive for AChR Abs (24%-49%) and GCs (10.7–15.6%) (15, 16). We found that there is a close relationship between the presence of GCs and the incidence of irAEs in TOMA patients. We propose that GCs may serve as biomarkers for irAEs in thymoma patients without MG. In addition, some autoimmune diseases, such as Hashimoto’s thyroiditis, systemic lupus, and Isaac’s syndrome, are closely related to thymoma (28). To date, whether thymic GCs exist in these thymoma-related AIDs other than MG remains unclear. The autoimmune regulator (AIRE), a key transcriptional regulator in medullary thymic epithelial cells (mTECs), is critical for central immune tolerance; its deficiency impairs the effective elimination of autoreactive T cells, relevant to multiple autoimmune diseases, including MG (29). As the core organ for T cell development and maturation, thymic immune microenvironment dysfunction is likely the main pathogenic basis for such autoimmune abnormalities. Based on this, we hypothesize that TOMA patients who are AChR Ab negative and have no other autoimmune diseases may represent a favorable population for ICI immunotherapy. The core value of this hypothesis lies in its ability to assess ICI treatment risk in thymoma patients based on clinical features (AChR Ab status, AID history) without the need for thymic GC detection. However, this hypothesis requires further validation through comprehensive autoantibody testing, systematic autoimmune disease screening, and large-scale cohort studies.

This study has several limitations because of its retrospective nature. MG is a rare autoimmune disease, and MGT patients with unresectable thymoma are exceptionally rare, leading to a modest sample size that limits the generalizability of some conclusions. MG flares and irAE types were documented on the basis of electronic medical records rather than standardized assessments by neurology specialists, potentially introducing data inaccuracies. Furthermore, given the potential heterogeneity of GC distribution in thymoma tissue, we recommend that future prospective studies prioritize intact thymic tissue from surgical resection to more comprehensively evaluate the association between GC distribution and irAEs. In addition, this was a single-center study, and the results may not be generalizable to other medical centers. We did not include thymoma patients with AIDs other than MG, which prevents us from verifying the cross-AID universality of the association between thymic immune dysregulation and irAEs. Integrating antibody titers, thymic histopathology, and changes in immune cell subsets may more effectively elucidate the mechanisms underlying ICI-induced MG exacerbation. Long-term follow-up after the occurrence of irAEs is necessary to further identify chronic irAEs and explore better treatment options.

## Conclusion

For MG patients with thymoma who receive one session of ICI therapy, close monitoring is recommended because of the high incidence of severe myocarditis and MC. Prophylactic maintenance of immunosuppressants prior to ICI initiation, combined with early steroid-IVIG dual therapy during irAE onset, appears critical for improving clinical outcomes. We propose that ectopic GCs may serve as a significant marker for selecting thymoma patients who are suitable candidates for ICI therapy. Further multicenter studies with standardized neurological assessments are warranted to validate these findings and refine patient selection criteria for immunotherapy in patients with thymomas.

## Data Availability

The original contributions presented in the study are included in the article/**Supplementary Material**. Further inquiries can be directed to the corresponding authors.
