# Supplementary material for: Immune-related adverse events in patients with preexisting myasthenia gravis and thymoma following immune checkpoint inhibitor treatment: a retrospective, observational study
Source: Front Immunol. 2026 Mar 10;17:1635001. doi: 10.3389/fimmu.2026.1635001 (PMC13008671; doi:10.3389/fimmu.2026.1635001)
Supplement: Supplementary file 1 [file Table1.docx]

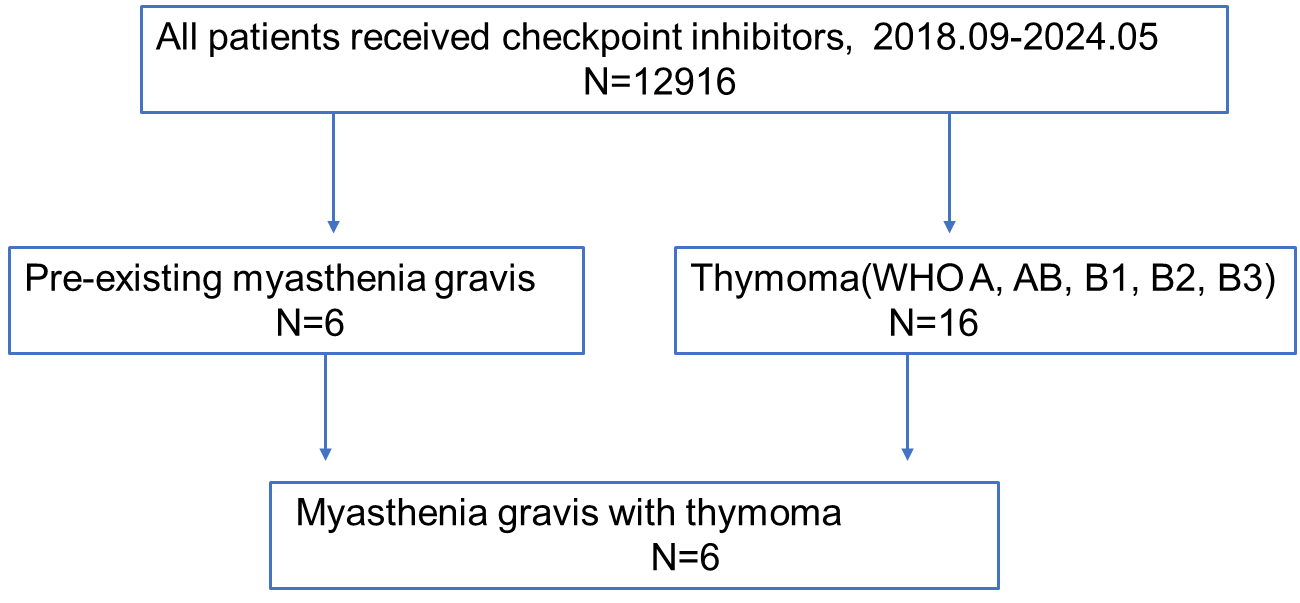


**Figure S1. Flow chart of participant inclusion in the study.**

**Table S1**. Clinical, diagnostic and outcome features of patients.

| **Patients ID** | **Age** | **Type of ICI** | **WHO** | **Masaoka** | **Reason for Unresectability** | **The type and grade of irAEs** | **Time to onset (days)** |
| --- | --- | --- | --- | --- | --- | --- | --- |
| NO 1 | 60s | PD-1 | B2 | IVb | Bone metastasis | Myasthenia gravis (4 grade), Myocarditis (3 grade), Anemia (2 grade) | 30 |
| NO 2 | 20s | PD-L1 | B2 | IVb | Bilateral lung metastases | Myasthenia gravis (4 grade), Myocarditis (3 grade), Thyroiditis (2 grade) | 21 |
| NO 3 | 40s | PD-1 | B2 | III | Invasion of the brachiocephalic vein and aorta | Myasthenia gravis (4 grade), Myocarditis (4 grade), Hepatitis (4 grade) | 7 |
| NO 4 | 40s | PD-1 | B1 | IVb | Pleural metastasis, Liver metastasis | Myasthenia gravis (4 grade), Myocarditis (5 grade), Hepatitis (3 grade), Thyroiditis (2 grade) | 3 |
| NO 5 | 60s | PD-1 | B2 | IVa | Pleural metastasis | Myasthenia gravis (4 grade), Myocarditis (5 grade), Hepatitis (4 grade), Thyroiditis (2 grade), Thrombocytopenia (3 grade), Anemia (1 grade) | 5 |
| NO 6 | 50s | PD-1 | B2 | IVa | Bilateral lung metastases | Myasthenia gravis (4 grade), Myocarditis (5 grade), Hepatitis (3 grade), Thyroiditis (2 grade) | 22 |
| NO 7 | 50s | PD-1 | B2 | IVa | Pleural metastasis | Hepatic failure (5 grade), Thyroiditis (2 grade) | 7 |
| NO 8 | 50s | PD-1 | B2 | IVa | Pericardial metastasis | Myocarditis (5 grade), Hepatitis (3 grade) | 5 |
| NO 9 | 30s | PD-1 | B3 | III | Invasion of the superior vena cava and aorta | Myocarditis (5 grade), Hepatitis (3 grade) | 21 |
| NO 10 | 50s | PD-1 | B3 | IVb | Bilateral lung metastases | Myocarditis (5 grade), Hepatitis (4 grade) | 13 |
| NO 11 | 50s | PD-1 | B2 | IVb | Pleural metastasis, Lymph node metastasis, Bilateral lung metastases | Skin rash (2 grade), Colitis (2 grade) | 7 |
| NO 12 | 70s | PD-1 | AB | III | Invasion of the brachiocephalic vein and aorta | None |  |
| NO 13 | 50s | PD-1 | B3 | IVa | Pleural metastasis | None |  |
| NO 14 | 50s | PD-1 | B2 | IVb | Invasion of the superior vena cava and brachiocephalic vein, Bilateral lung metastases | None |  |
| NO 15 | 40s | PD-1 | B2 | III | Pulmonary invasion | None |  |
| NO 16 | 50s | PD-1 | B3 | IVa | Pericardial invasion, Pulmonary lobe invasion, Lymph node metastasis | None |  |

**Table S2**. The relationship between GCs and irAEs.

| **Patients ID** | **Pre-existing MG** | **irAEs grade** | **Number of GC in thymic** | **Recurrence after thymoma resection** | **Thymoma puncture tissue** | **Thymoma tissue** |
| --- | --- | --- | --- | --- | --- | --- |
| NO 1 | Yes | 4 | NA | No | Yes | No |
| NO 2 | Yes | 4 | 11 | Yes | No | Yes |
| NO 3 | Yes | 4 | NA | Yes | No | No |
| NO 4 | Yes | 5 | 0 | Yes | No | Yes |
| NO 5 | Yes | 5 | 2 | Yes | No | Yes |
| NO 6 | Yes | 5 | 1 | Yes | Yes | Yes |
| NO 7 | No | 5 | NA | No | Yes | No |
| NO 8 | No | 5 | 6 | No | No | Yes |
| NO 9 | No | 5 | NA | No | Yes | No |
| NO 10 | No | 5 | NA | No | Yes | No |
| NO 11 | No | 2 | 4 | Yes | No | Yes |
| NO 12 | No | 0 | 0 | No | No | Yes |
| NO 13 | No | 0 | 0 | Yes | No | Yes |
| NO 14 | No | 0 | NA | No | Yes | No |
| NO 15 | No | 0 | NA | No | Yes | No |
| NO 16 | No | 0 | NA | No | Yes | No |

NA: Not applicable
